# Supplementary figures and images for: YeeD is an essential partner for YeeE-mediated thiosulfate uptake in bacteria and regulates thiosulfate ion decomposition
Source: PLoS Biol. 2024 Apr 24;22(4):e3002601. doi: 10.1371/journal.pbio.3002601 (PMC11073785; doi:10.1371/journal.pbio.3002601)

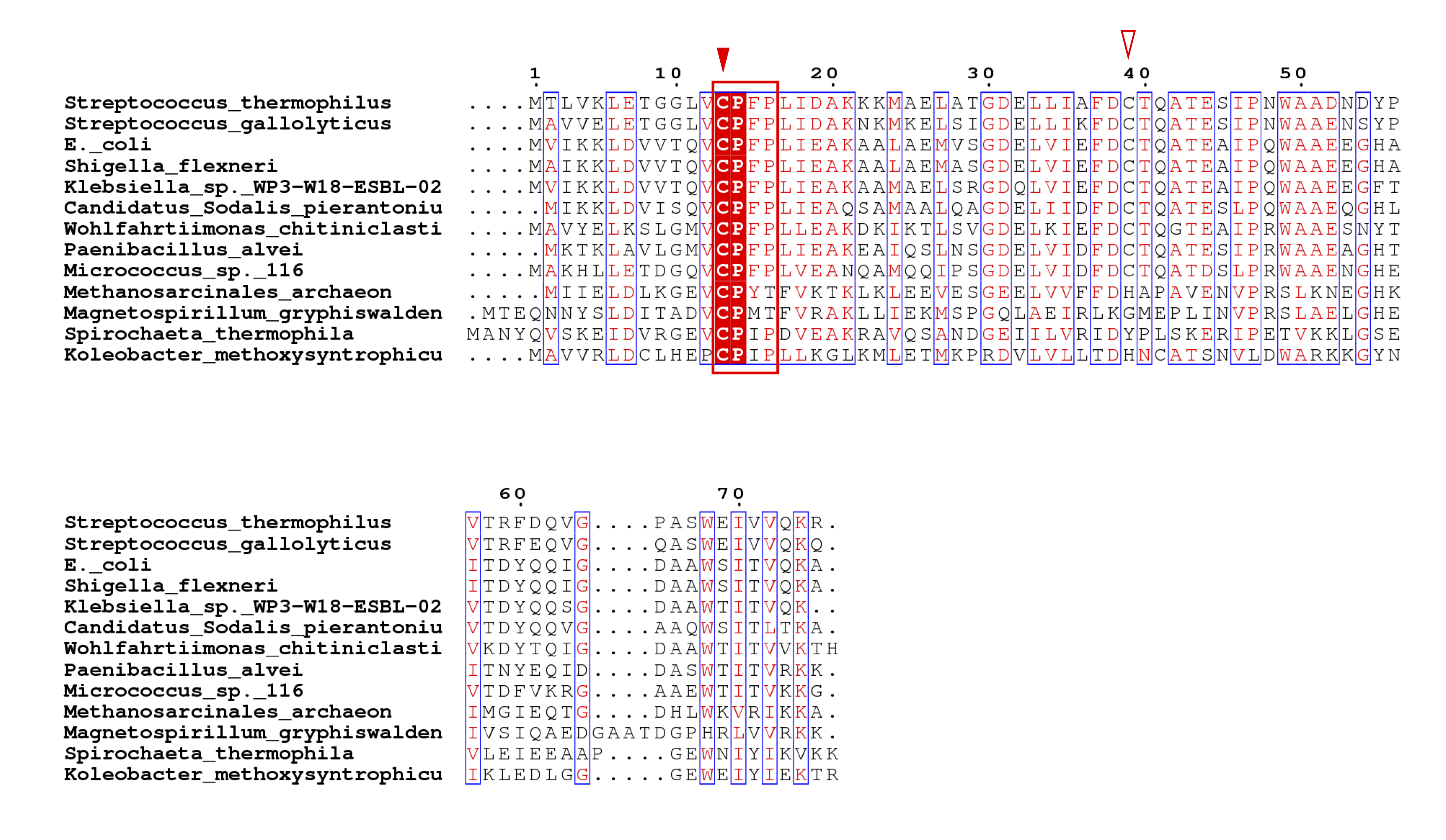

Supplement: S1 Fig — The UniProt IDs of YeeD sequences used are as follows: E. coli YeeD, P33014; Spirochaeta_thermophila YeeD, G0GAP7; Candidatus Sodalis pierantonius YeeD, W0HL40; Klebsiella_sp._WP3-W18-ESBL-02 YeeD, A0A7I6Q8F8; Methanosarcinales archaeon YeeD, A0A822J3Z6, Streptococcus thermophilus YeeD, A0A8D6XUG1; Koleobacter methoxysyntrophicus YeeD, A0A8A0RNL6; Wohlfahrtiimonas chitiniclastica YeeD, L8Y0N6; Paenibacillus alvei YeeD, A0A383RJV0; Shigella flexneri YeeD, A0A384L8W9; Streptococcus gallolyticus YeeD, A0A380K504; Micrococcus_sp._116 YeeD, A0A653IT90; Magnetospirillum gryphiswaldense YeeD, V6F4H7. The red rectangle indicates the CPxP motif. The first and second cysteine residues (solid and open red arrowheads) are completely and not completely conserved among species, respectively. The figure was generated using ESPript 3.0 [45]. (TIFF) [file pbio.3002601.s001.tiff]

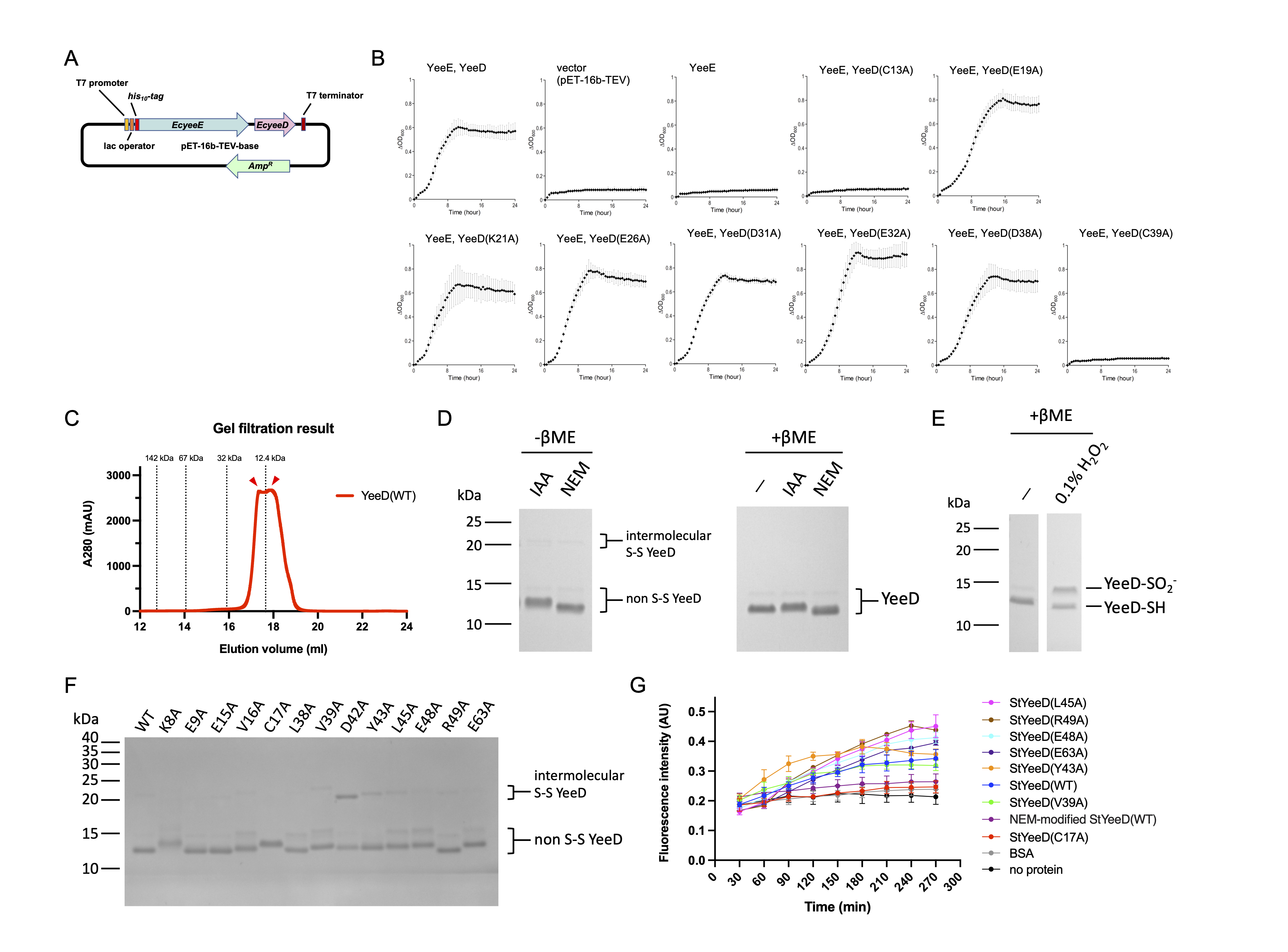

Supplement: S2 Fig — (A) Details of plasmid (pAZ061), used as the positive control for the growth complementation assay. Tandemly located EcyeeE and EcyeeD are regulated by the same promoter. A His10-tag is attached to the N-terminal side of EcYeeE. Based on pAZ061, a deletion and several point mutations on EcyeeD were introduced. (B) Original data from the growth complementation assay in Fig 2C. Error bars represent the SD of 3 measurements. (C) Gel filtration profile of purified StYeeD(WT), which eluted with 2 peaks (red arrowheads). The eluted positions of standard proteins and their molecular masses are shown. (D) Nonreducing (−βME) and reducing (+βME) SDS-PAGE of StYeeD after iodoacetamide (IAA)- or N-Ethylmaleimide (NEM) treatment. Only minor fractions show intermolecular disulfide bond formation between StYeeDs(WT). (E) Irreversible oxidation of StYeeD by hydrogen peroxide. Before reducing SDS-PAGE, the hydrogen peroxide treatment was performed. (F) Nonreducing SDS-PAGE profile of StYeeD mutants after NEM treatment. (G) Original data for the enzymatic activity of StYeeD detected by HSip-1 in Fig 2I. Error bars show the SD from 3 measurements. The underlying data for (B), (C), and (G) can be found in S1 Data. The original gel images of (D–F) can be found in S1 Raw Images. (TIFF) [file pbio.3002601.s002.tiff]

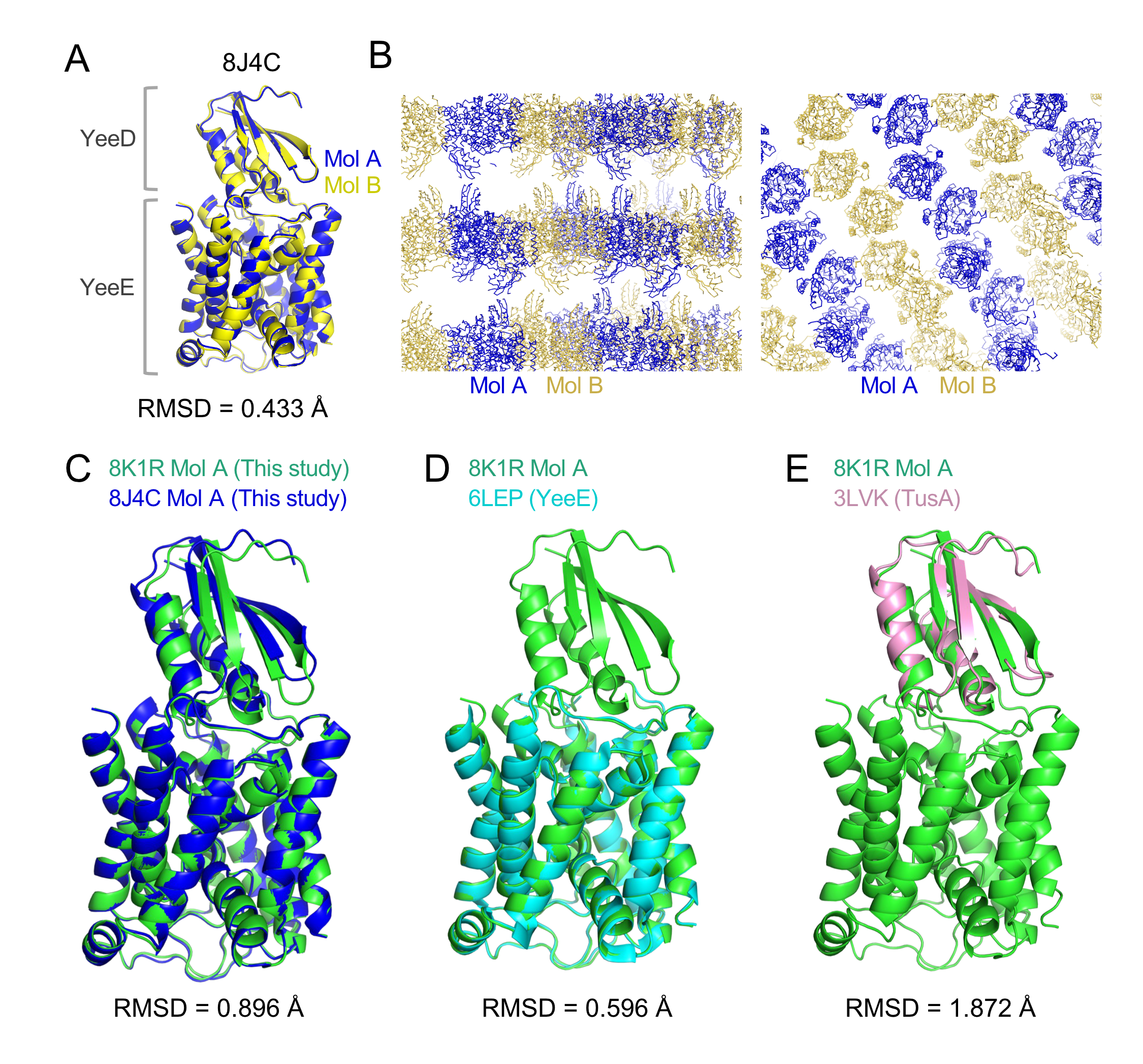

Supplement: S3 Fig — (A) A comparison of the crystal structures Mol A and Mol B in the asymmetric unit of 8J4C. (B) Crystal packing of 8J4C viewed from 2 different directions. (C) Comparison of the crystal structures of Mol A of 8K1R and MolA of 8J4C. (D and E) Comparisons of the crystal structures of StYeeE-YeeD complex (MolA) and StYeeE (PDB ID 6LEP) (D) or E. coli TusA (PDB ID 3LVK) (E). (TIFF) [file pbio.3002601.s003.tiff]

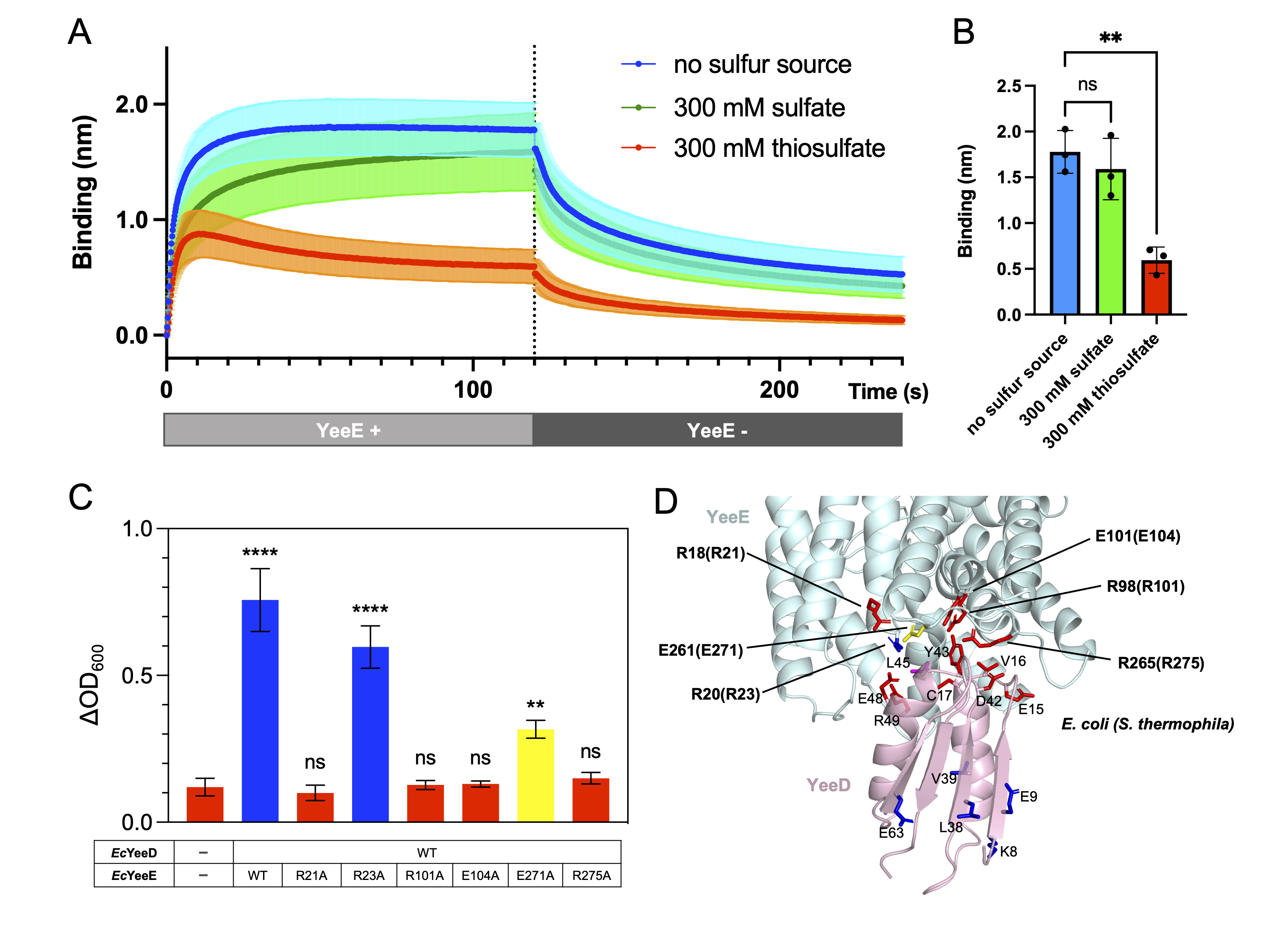

Supplement: S4 Fig — (A) Real-time detection by the BLI method of association and dissociation of StYeeE with/from solidified StYeeD in buffer without a sulfur source, with 300 mM Na2SO4, or with 300 mM Na2S2O3. Each line shows the mean value of 3 measurements with the SD. The dashed line indicates the 120-s point. (B) Comparison of mean values of StYeeE binding to solidified StYeeD at 120 s. Error bars represent the SD of 3 measurements. Statistical significance compared with no sulfur source was determined using one-way analysis of variance (ANOVA) followed by Dunnett’s multiple comparisons tests (**, p < 0.01; ns, not significant). (C) Growth complementation assay of ΔcysPUWAΔyeeE (DE3) cells, depending on EcYeeE and EcYeeD expressed from plasmids. Mean values of increased OD600 (ΔOD600) after 24 h are shown. Error bars represent the SD from 3 measurements. Statistical significance compared with the ΔcysPUWA ΔyeeE (DE3) cells possessing an empty vector was determined using one-way ANOVA followed by Dunnett’s multiple comparisons tests (****, p < 0.0001; **, p < 0.01; ns, not significant). The underlying data for (A–C) can be found in S1 Data. (D) Mutation site mapping on the crystal structure of StYeeE-YeeD complex (PDB ID 8J4C). The side chains of amino acid residues of StYeeE corresponding to those of EcYeeE mutated in (C) are shown as stick models with the same colors as in (C). The amino acid residues of StYeeD shown in Fig 4C are also indicated. (TIFF) [file pbio.3002601.s004.tiff]

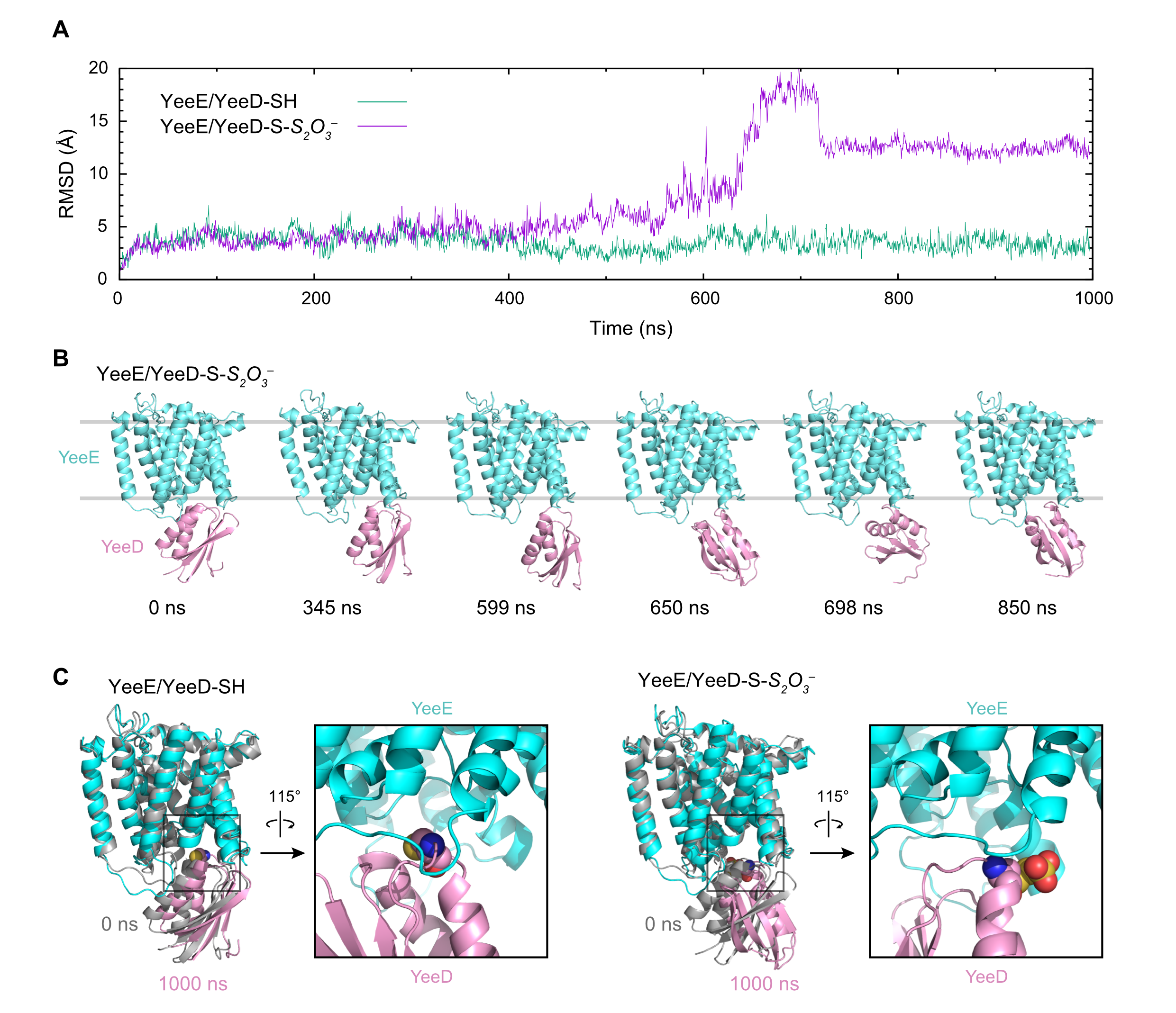

Supplement: S5 Fig — (A) Time courses of the Cα-RMSD calculated for StYeeD regions relative to the initial structures. For the RMSD calculations, each StYeeE region was superimposed. (B) Snapshots in the MD simulation of StYeeE/YeeD-S-S2O3-. (C) Comparison between the initial structure (0 ns) and final snapshot (1,000 ns) for StYeeE/YeeD-SH (left) and StYeeE/YeeD-S-S2O3- (right). The square boxes display magnified views of the region around C17 (spheres). The underlying data can be found in S2 Data. (TIFF) [file pbio.3002601.s005.tiff]

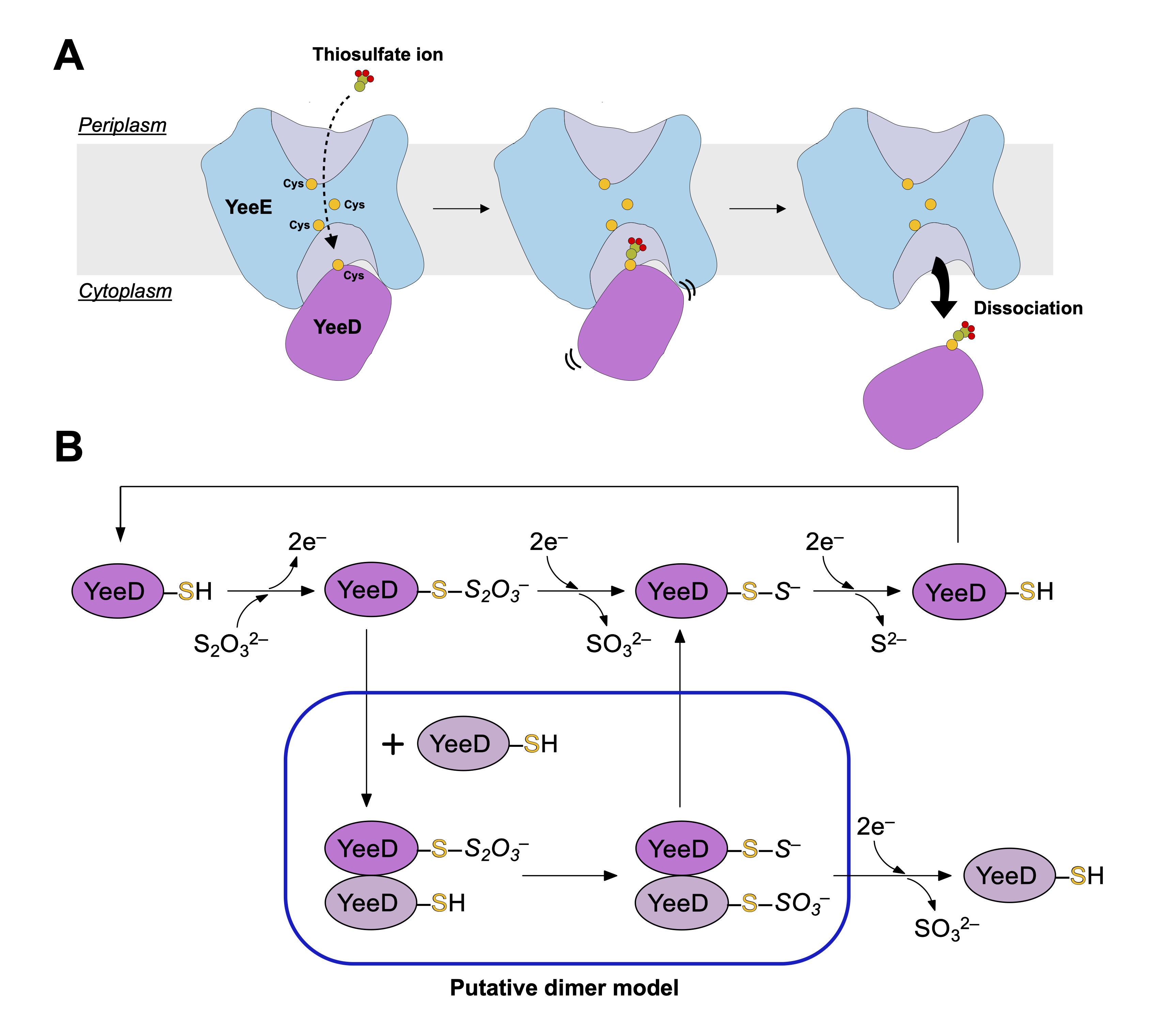

Supplement: S6 Fig — (A) Uptake of a thiosulfate ion by YeeE and YeeD. The thiosulfate ion is relayed by conserved cysteine residues of YeeE and binds to the conserved cysteine residue of YeeD. After that, the association of YeeD with YeeE is destabilized. YeeD then dissociates from YeeE and decomposes the thiosulfate. (B) Thiosulfate decomposition by YeeD. First, thiosulfate binds directly to the conserved cysteine residue of YeeD. Next, sulfide ion (SO32-) is released. Finally, sulfite ion (S2-) is released. In the blue box, a putative dimerization model of StYeeD is shown. (TIFF) [file pbio.3002601.s006.tiff]

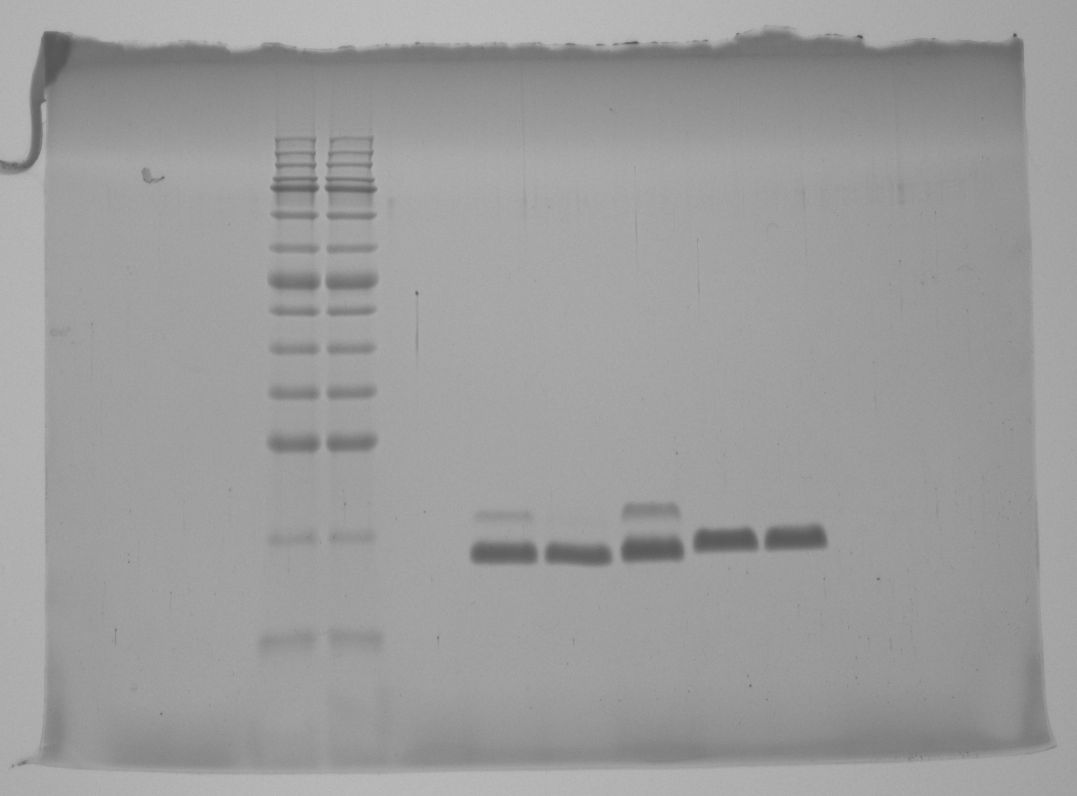

Supplement: S1 Raw Images — (ZIP) [file pbio.3002601.s009.zip › S1_raw_images/S1_Raw_Images_Fig2DE/StYeeD_WT_C17A_MS.Tif]

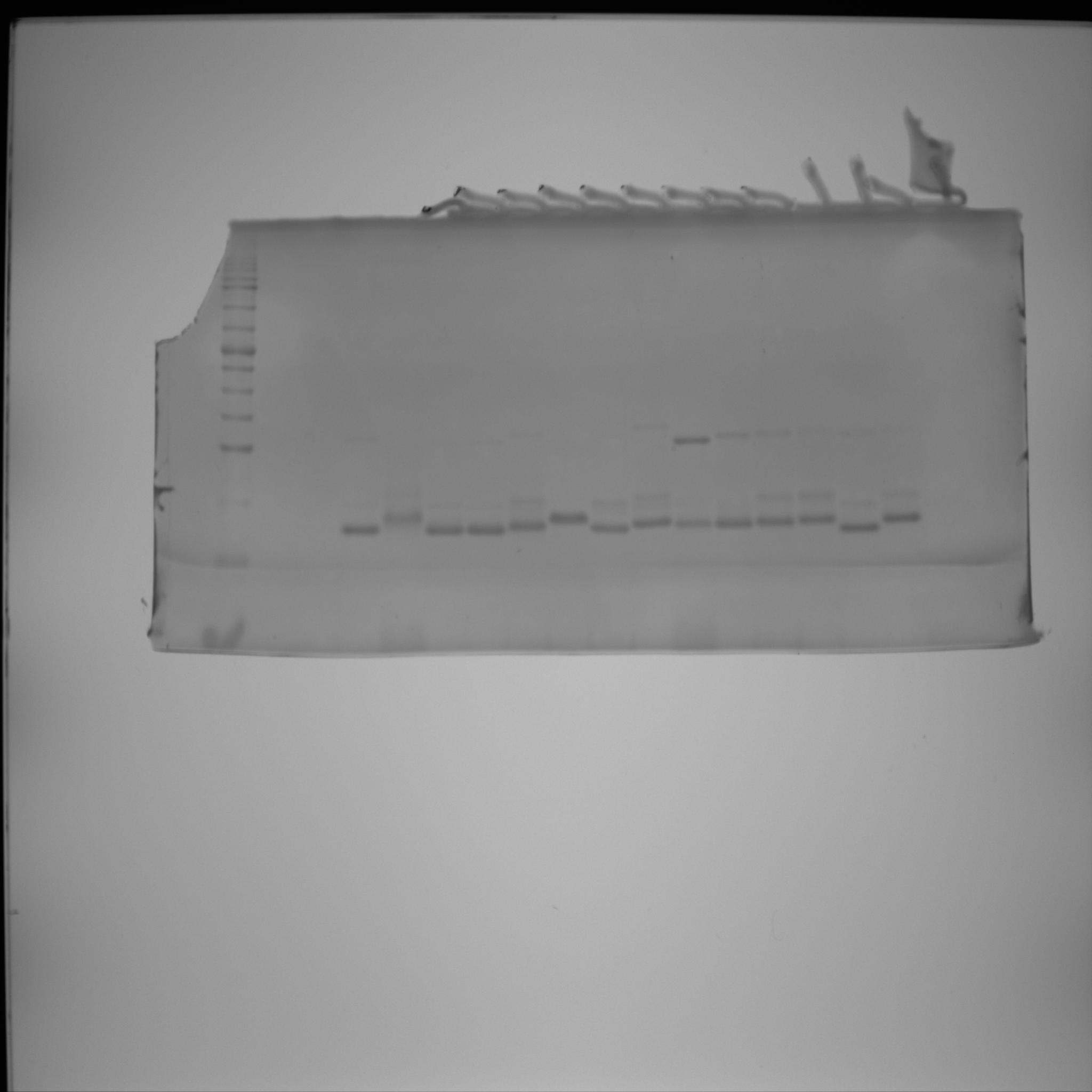

Supplement: S1 Raw Images — (ZIP) [file pbio.3002601.s009.zip › S1_raw_images/S1_Raw_Images_FigS2D-F/FigS5F.Tif]

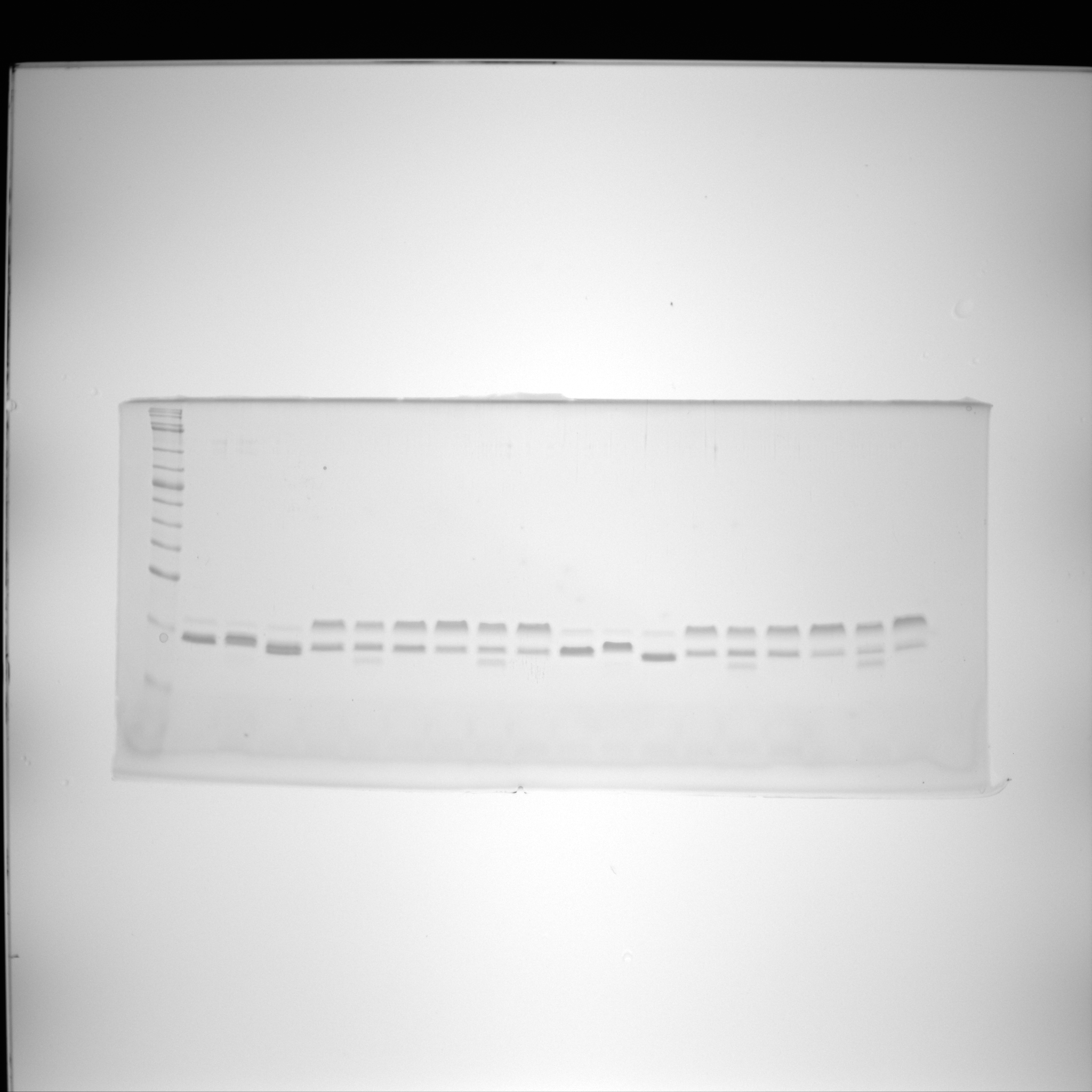

Supplement: S1 Raw Images — (ZIP) [file pbio.3002601.s009.zip › S1_raw_images/S1_Raw_Images_FigS2D-F/FigS2E.Tif]

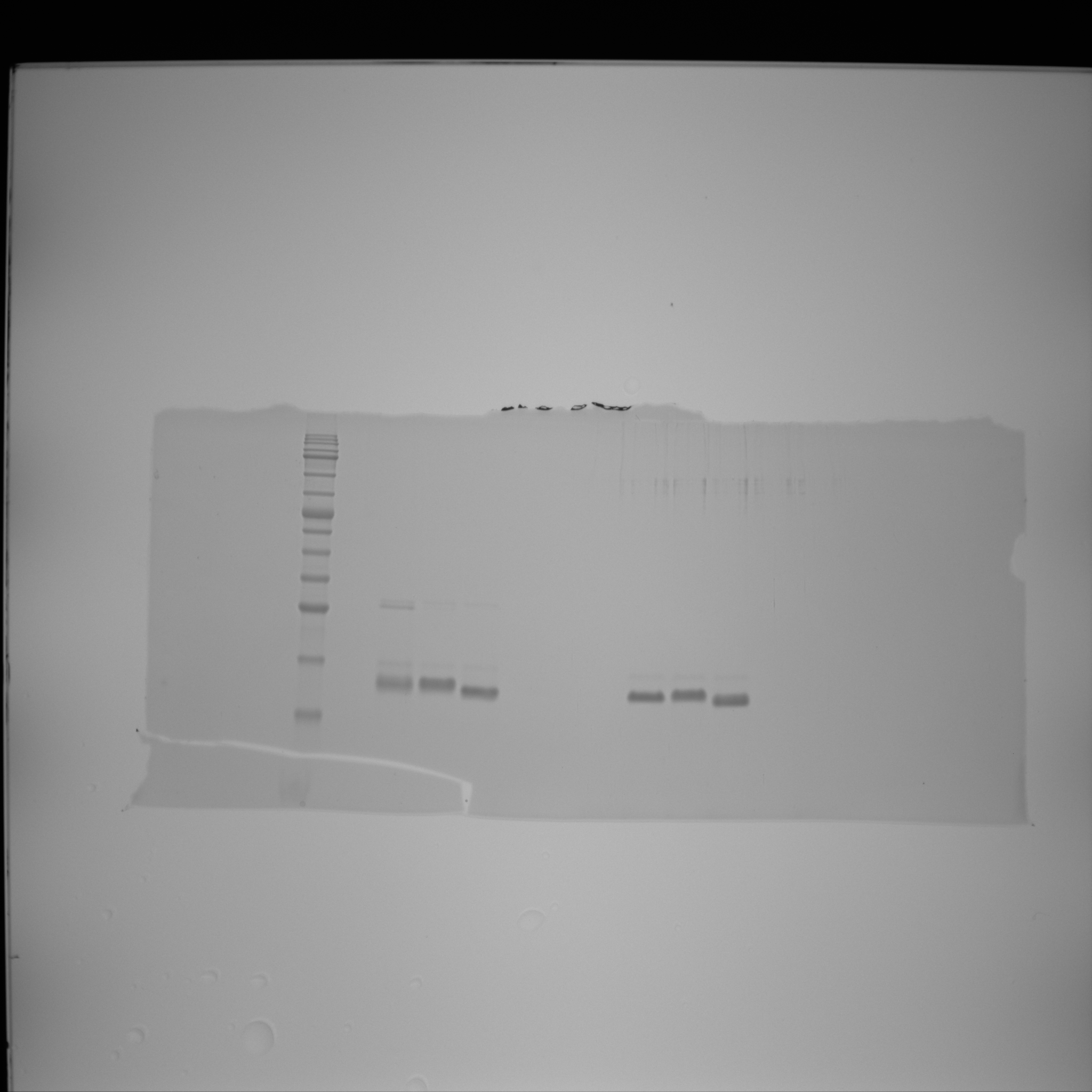

Supplement: S1 Raw Images — (ZIP) [file pbio.3002601.s009.zip › S1_raw_images/S1_Raw_Images_FigS2D-F/FigS2D.Tif]
