# Supplementary material for: YeeD is an essential partner for YeeE-mediated thiosulfate uptake in bacteria and regulates thiosulfate ion decomposition
Source: PLoS Biol. 2024 Apr 24;22(4):e3002601. doi: 10.1371/journal.pbio.3002601 (PMC11073785; doi:10.1371/journal.pbio.3002601)
Supplement: Attachment — Submitted filename: full-revision_tt3.pdf [file pbio.3002601.s011.pdf]

Full Revision

Review COMMONS

Manuscript number: RC- 2023-02150
Corresponding author(s): Muneyoshi Ichikawa and Tomoya Tsukazaki

1. General Statements [optional]

We sincerely appreciate the reviewers' comments on our manuscript. Based on their valuable feedback, we have re-conducted several experiments to enhance the quality of our paper and undertaken substantial revisions. Moreover, during this revision period, we performed molecular dynamics simulations of the YeeE-YeeD complex (line 300-314 and Fig. S5) to strengthen the reliability of our model in Fig. S6A (line 363-364). This addition ensures that our research findings are more robust and compelling for our readers. The resubmitted paper incorporates responses to the reviewers' comments (see below).

2. Point-by-point description of the revisions

> *---*
> *Reviewer #1 (Evidence, reproducibility and clarity (Required)):*
>
> *The manuscript develops the authors' previous work on the structure of the YeeE protein by presenting a co-structure with YeeD and investigating the role of certain key cysteine residues, especially C17 of YeeD. To this reviewer an entirely plausible mechanism for YeeD/E co-ordinated transport of thiosufate through the membrane and cleavage to sulfide and sulfite which are released into the cytoplasm is proposed on the basis of functional studies. The work is clearly described, the crystallography stats look good.*

Thank you very much for your highly positive comments. We sincerely appreciate them.

> *Major comment: The 'cysteine relay' followed by a key role for C17 of YeeD in releasing a sulfide looks very plausible and makes the work of more general interest. An aspect that is not addressed is that of energetics. Moving thiosulfate into the cytoplasm as sulfide and sulfite means apparently that two negative charges net are generated in the cytoplasm for each thiosulfate taken up. This seems too simplistic (protons released as the bound sulfite is released b hydrolysis) but if thiosulfate were to be moved the whole way across there would be a divalent anion uniport which would work against the membrane potential negative inside (ie the main component of the protonmotive force). There is no mention in the paper of any pmf dependence and presumably the structure of YeeE shows no evidence of putative proton pathways? Some discussion of this and any wider implications could enhance the paper. In some ways the proposed transport scheme has some resemblance to Mitchells's old group translocation proposal for transport.*

Thank you for highlighting the significance of the 'cysteine relay.' We also believe that this aspect is likely to interest a broad readership. Regarding protons, YeeE does not have apparent proton pathways inside, and we currently do not have data on its dependence on the pmf. Investigating pmf dependence falls beyond the scope of this study, hence we plan to explore this in future research. We appreciate you for pointing out that the YeeE-YeeD is a reminiscence of Mitchell's

Review COMMONS

original proposal of group translocation. This is a very intriguing point, and we have now included a discussion of this, along with a relevant citation, in the Discussion section (lines 356-357).

> *Reviewer #1 (Significance (Required)):*
>
> *The subject of thiosulfate transport (movement) into bacteria is arguably of interest only to a narrow group of bacterial biochemists. However, the contents of this manuscript ought to be of wider interest because the YeeD/E system described is unusual in doing more that catalysing transport alone. Whether the authors' description in their title of 'sophisticated' is an appropriate adjective I am not sure. The term 'cofactor' applied to YeeD seems 'odd' to this reviewer. It is not a cofactor in the usual sense eg NADH.*

We appreciate your comments. We have modified the title and avoided the unsuitable word 'cofactor' to describe YeeD.

> *reviewer's expertise: bactrial energetics but little knowledge of sulfur metabolism*
>
> *Reviewer #2 (Evidence, reproducibility and clarity (Required)):*
>
> *Summary:*
> *The publication "Structure and function of a YeeE-YeeD complex for sophisticated thiosulfate uptake" by Ikei et al. shows the protein-protein interaction of a thiosulfate transporter YeeE and a sulfur transferase YeeD, a TusA-family protein. The transporter YeeE has been structurally characterized previously, without showing its functional activity in a purified reconstituted system. This experiment complementing the previous publication is provided here, furthermore proving the functionality of the transporter. These experiments were further extended by the characterization of the cytoplasmic acceptor protein. This acceptor was proven to be YeeD, by structural characterization and biolayer interferometry. The binding kinetics between YeeD and YeeE were measured, quantifying the binding affinity between the two proteins. Furthermore, the surface residues of YeeD were specified by amino acid exchange mutants. Thus, the structure and essential residues were characterized protein. The interaction of sulfur transferase YeeD with the thiosulfate transporter YeeE is a novelty to the field. This illuminates the first time a specific function of YeeD in thiosulfate assimilation.*

We appreciate your positive review and for recognizing the significance of our work in uncovering the functions of the YeeE and YeeD complex. We have addressed the following major and minor comments, thereby improving our manuscript. We appreciate the your constructive feedback.

> *Major comments:*
> *I see the following major problem: The YeeD protein preparations used in the experiments contained several different protein species. Mass spectrometry showed the existence of the monomeric reduced protein, a TusA sulfinate and a TusA thiosulfonate. There is obviously an oxidation of cysteine to cysteine sulfinate, possibly due to the presence of oxygen as shown in Fig. 2D and stated in the text. The formation of sulfinates has to be avoided. This can be achieved by the use of stronger reducing agents or by purification under strict exlusion of oxygen. The formation of sulfenic, sulfinic and sulfonic acid on cysteines by oxidation has been reviewed by Ezraty et al 2017 Nat Rev Microbiol.*

To answer these points, we have extensively several experiments and analyses, and modified the text. In the mass spectrometry analysis of purified *St*YeeD, three major peaks are observed (Fig. 2D), but they do not necessarily reflect actual relative abundances due to the nature of mass spectrometry analysis. Therefore, we also analyzed the purified *St*YeeD by non-reducing SDS-PAGE, which showed very few molecular species with S-S bonds, with over 90% existing as YeeD-SH (Fig. S2D). We considered this level of purity sufficient for conducting biochemical analyses. Furthermore, although a small amount of YeeD-SO_2^- was observed, this would be inactive and thus not impact the activity of StYeeD because a similar irreversible modification product, NEM-modified StYeeD(WT), was inactive (Fig. S2G).
We have also provided non-reducing SDS-PAGE results for each mutant StYeeD in Fig. S2F. All StYeeD mutants except for L45A showed a similar pattern to StYeeD(WT). Conducting experiments under anaerobic conditions is quite challenging in our laboratory facility, so we have displayed non-reducing SDS-PAGE profiles of all proteins used in order to avoid misunderstanding. We have also tried the purification in the presence of DTT, a stronger reducing agent, but the fraction of YeeD-SO_2^- was not significantly changed.
In the revised version, mass spectrometry analyses were reperformed using DTT-reduced YeeD, resulting in more precise data (Fig. 2D–H). Based on these results and your valuable comments, we have rewritten the paragraph entitled '**Thiosulfate decomposition activity of YeeD and its catalytic center residue**' to represent the reduction/oxidation forms accurately. We have also cited the *Nat. Rev. Microbiol.* review in the text (line 185).

> *In their in vitro assays, the authors use exceptionally high thiosulfate concentrations of 300 mM. This is so far from any physiologically relevant concentrations that strong doubt is shed the validity of any conclusions transferred from the in vitro to the in vivo situation.*

In the revised version, the mass spectrometry analysis was reperformed with a thiosulfate concentration of 500 μM, which is the same concentration of thiosulfate used in the thiosulfate decomposition experiments. To clarify this, we have included the thiosulfate ion concentrations in the legend of Fig 2.

> *L247 and Fig5: The proposed mechanism cannot be true. Binding of thiosulfate to a reduced TusA protein is not possible without release of electrons. Where do these electrons go? In the proposed scheme, the number of electrons before and after the reaction steps is not equal (Fig. 5). A release of the sulfur atom between the cysteine sulfur atom and the oxidized sulfur atom is impossible.*

Thank you for your insightful comments. We have revised Fig. S6B to represent a better model. However, elucidating the electron pathway falls outside the scope of this study, and we cannot offer a definitive explanation. We have addressed this limitation in the *Discussion* section and highlighted it as a topic for future research.

> *Have the authors checked whether TusA dimers are formed via disulfide bridges? If so, thiosulfate could resolve these disulfides leading to reduced TusA and thiosulfonated TusA (YeeD-S-S-YeeD + S2O32- → YeeD-S-S-SO3- + YeeD-S-).*
> *It cannot be excluded that the YeeD-S-SO3- species is a result of removal of sulfite from the YeeD-S-S2O3- species (possibly by transfer to another YeeD molecule) resulting in YeeD-*

Review COMMONS

> *S-S- oxidized by molecular oxygen to YeeD-S-SO3-.*

Upon answering to this comment, we have re-examined the gel filtration result using gel filtration markers. We found that a fraction of YeeD exists as dimers in solution, as shown in Fig. S2C. By performing non-reducing SDS-PAGE, it was shown that these YeeD dimers were not due to intermolecular disulfide bond (Fig. S2D). Following your valuable suggestion, we have introduced the possibility that YeeD can function as a dimer into our model, as presented in a box in Fig. S6B.

> *Sulfide may be formed by a reaction of YeeD-S- with S2O32- to YeeD-S-SO3- and S2- or reaction of YeeD-S-S- with S2O32- to YeeD-S-S2O3- and S2-. As there is the formation of sulfinic acid that prevents clear conclusions, I suggest repeating the experiments on thiosulfate decomposition under anaerobic conditions to clarify the reaction mechanism. Anoxic buffers and strong reducing agents may prevent chemical oxidation.*

As described above, based on the non-reducing SDS-PAGE results (Fig. S2D), we believe that the low presence of oxidized species does not significantly affect our analysis. Moreover, the mass spectrometry analysis after DTT treatment yielded more precise results (Fig. 2D–H). As noted above, conducting experiments under anaerobic conditions is challenging in our facility, so we kindly request your understanding and consideration of the revisions made in this manuscript.

> *Minor comments:*

In response to the minor comments, we have revised the manuscript.

> *L58 What is the nature of the binding of the thiosulfate ion during the transport via YeeE. Is it covalently bound? Please comment in the text.*

In our previous study (Tanaka et al., *Sci. Adv.*, 2020), we proposed that thiosulfate ions were transported via hydrogen bonds. Responding to your comment, we have included the explanation in the text and cited Tanaka et al., 2020 (lines 66-67).

> *L76-L77 Is there a publication on the functionality of the Corynebacterium YeeD-YeeE fusion? The term "cofactor" does not apply to YeeD, which is a 9-kDa protein.*

Since the function of *Corynebacterium* YeeD-YeeE has not been reported, we have changed the sentence to "In some bacteria, such as Gram-positive *Corynebacterium* species, YeeE and YeeD are encoded as one polypeptide." We have also avoided the word "cofactor" in the revised text (lines 89-91).

> *L114 YeeD was probably accidentally lowercased here as Yeed*

We have corrected this error (line 134).

> *L119 Please specify what the negative control consisted of.*

We have elaborated on the conditions (lines 140-141).

> *L120-122 In Fig 2c, the mutations E19A, K21A, E26A, D31A, E32A and D38A are still shown, but an explanation or description of the results is missing. The reason for investigation of these mutations should be stated in the text.*

We have added the requested mutation information (line 146).

> *L137 If thiosulfate was not added before the MALDI-TOF, where did the sulfonate S-SO3 originate from? Is this an artifact formed during the heterologous production or purification? Please comment on this possibility in the text.*

We think that the -S-SO_3^- form arose during purification (Fig. 2D). The -S-SO_3^- form disappeared upon reduction by DTT (Fig. 2F). It is possible to consider it as an intermediate state in the catalytic cycle of YeeD. We commented on this in the section entitled "**Thiosulfate decomposition activity of YeeD and its catalytic center residue**."

> *L144 Please state in the text whether these experiments were performed under aerobic or anaerobic conditions. The sulfinic acid is likely a product of a spontaneous chemical reaction with molecular oxygen.*

Thank you for your feedback. We have now included information about the aerobic conditions in the main text (line 166-167) and added comments regarding the mass spectrometry results at the end of the paragraph (lines 191-201).

> *L148 It should be stated in the text whether YeeD in Fig2G was reduced with DTT as in Fig 2F or non-reduced as in Fig. 2D before thiosulfate was added. Only the reduced YeeD can yield conclusive results on the loading with sulfur, as there is already a thiosulfonate bound to the protein after purification.*

Thank you for pointing this out. For mass spectrometry analysis, data were re-obtained, and DTT-treated sample was used for the thiosulfate condition in this revised version. Furthermore, we performed mass spectrometry analysis for the hydrogen peroxide condition using DTT-treated sample. Figures were replaced with revised ones (Fig. 2D–H). The text in the section "**Thiosulfate decomposition activity of YeeD and its catalytic center residue**" was appropriately re-written. Detailed sample preparation is also described in *MATERIALS AND METHODS* section.

> *L154 The YeeD used for measurement of sulfide formation must be reduced before the experiments. It is not stated in the text if this is the case. Also, the release of sulfide requires electrons. It should be commented where these electrons originate from.*

The sample in the purification process contains β-ME until just before the final column (gel filtration). As shown in Fig. S2D, more than 90% of the purified product is in a reduced state after gel filtration. For mass spectrometry analysis, data were re-obtained using DTT-treated samples, and the figures were replaced with new ones (Fig. 2D–H). Binding and activity measurements were conducted in the presence of β-ME. To avoid the confusion of the readers, the buffer conditions were included in the legends of both Fig. 2 and Fig. 4, along with the details in the *MATERIALS AND METHODS* section. Regarding electron origin, since the electron route remains unknown at this stage, we have added the explanation as a sentence in the *Discussion* section (lines 370-372).

> *L159-160 If the mutation of the non-conserved YeeD cysteine inhibits growth, can anything be said about its function?*

Regarding the non-conserved Cys in EcYeeD, we added some sentences in the *Discussion* section (lines 393-397)

L214 Is it possible to provide the Kd and KD values for the mutant proteins?

The k_a, k_d and K_D values the interactions between YeeE and YeeD proteins have been provided in Table 2. To provide these values for all the YeeD derivatives, the data was re-analyzed, and therefore, the value of the WT YeeD is slightly different from the original manuscript.

L229 Stating a need of YeeD for thiosulfate uptake by YeeE is somewhat misleading as thiosulfate was also imported into liposomes by YeeE alone. Maybe state that YeeD is a required component for growth when thiosulfate is imported via YeeE.

We have addressed the incorrect wording (lines 317-318).

Reviewer #2 (Significance (Required)):

The work of Ikei and colleagues significantly advances our understanding of thiosulfate import in Escherichia coli (E. coli) and prokaryotes in general. Sulfur metabolism as a field is generally considered to be underexplored, with a notable lack of biochemical and structural information on membrane transporters responsible for the movement of both inorganic and organic sulfur compounds. The mechanisms involved in sulfur transport are also relatively poorly understood.

The proteins of the TusA family in E. coli exhibit distinct functions, although the precise function has only been determined for the canonical and namesake protein TusA. The discovered genetic evidence and the interaction of YeeE and YeeD adds significantly to our understanding of sulfur transfer reactions.

The novelty of this reaction is of particular interest to researchers studying prokaryotic physiology, especially the synthesis of sulfur-containing cofactors such as coenzyme A (CoA), biotin, lipoate, thiamine, and iron-sulfur (FeS) clusters, as well as the biosynthesis of cysteine and methionine. In addition, recent findings related to the TusA family protein YeeD elucidate a novel mechanism for sulfur mobilization and transfer that will be of interest to researchers involved in the regulation of sulfur metabolism, sulfur dissimilation, and ecological studies focused on sulfur utilization. Thus, a wide range of studies could be influenced by this review. Areas of expertise include dissimilatory sulfur oxidation, sulfur transfer reactions, and protein-protein interactions.

Thank you again for emphasizing the importance of our work. We also believe this study significantly advances the understanding of thiosulfate import in prokaryotes, shedding light on the underexplored field of sulfur metabolism. This has implications for various areas of study.

Reviewer #3 (Evidence, reproducibility and clarity (Required)):

The manuscript "Structure and function of a YeeE-YeeD complex for sophisticated thiosulfate uptake" by Ikei et al., reports the enzymatic characterization, transport capability and concerted function of YeeE and YeeD. Moreover, the authors report the crystal structures of two mutant variants of the complex.

The present work fills an important gap in understanding thiosulfate uptake and the individual roles of the YeeE and YeeD proteins in this process. This Reviewer believes that the paper has the potential of becoming an important reference in the field. However, this Reviewer has two or three major comments, besides a couple of minor ones, that would like the authors to address.

Full Revision

We appreciate your valuable comments. We have addressed both major and minor comments in our revisions, improving our manuscript.

> *This Reviewer hypothesizes that some of the comments might derive from a poor understanding of the text, derived from the way the manuscript is written. So, this Reviewer urges the Authors to take these comments as positive feedback, and build on these to improve the manuscript (namely on English and grammar).*

We have diligently revised the manuscript, addressing your major concerns related to sulfide terminology and explanations in crystal structure analysis as below. These revisions have enhanced clarity, and a native English speaker has reviewed and refined our text for language and grammar.

> *MAJOR CONCERNS*
> *1. There is no clue on the title and, more importantly, on the Abstract, to which microorganism the Authors are reporting this work. Only later one we are introduced to Spirochaeta thermophila, but this information should be front and center (at least in the Abstract);*

We recognize the importance of clearly indicating the microorganism in our work. In accordance with the comments, we have revised both the title and Abstract, ensuring that the species is clearly identified in the Abstract.

> *2. Also, in the Abstract, the Authors only mention the 2.6 A resolution structure, leaving behind the 3.34 A one. This becomes very confusing, especially once one gets to the Results section (more comments below);*

We apologize for any confusion arising from the omission of the 3.34 A resolution structure in the Abstract. In the revised Abstract, we have included both the 2.60 A and 3.34 A resolution structures. As per your suggestion, we have also provided detailed information about the determination of these structures in the *Results*, minimizing potential confusion for readers (lines 217-233).

> *3. The Authors mention in line 137 and Fig. 2D that a "sulfonate" moiety is formed at C17. However, cysteine sulfonation is an irreversible process, so how would the enzyme recover from this modification to allow turnover of the mechanism?;*

We apologize for the poorly written passage that led to confusion. This paragraph has been revised with the appropriate wording and a proper mention of the reduction and oxidation of the -SH group. We now use the appropriate terms, such as sulfinic acid (-S-O_2^-), sulfonic acid (-S-O_3^-), and perthiosulfonic acid (-S-SO_3^-) to describe the sulfur-related modification states. In contrast to sulfonic acid (-SO_3^-) formed by the oxidization of the cysteine residue that is an irreversible process, perthiosulfonic oxidization of cysteine residue (-S-SO_3^-) is a reversible process, as shown in (E. Doka *et al.*, *Sci Adv* **6**, eaax8358 (2020)). Therefore, the modified YeeD molecules should be able to recover to the original state.

> *4. If the "sulfonylation" reported in line 137 and Fig. 2D is not a sulfonylation of the cysteine (because the peak disappears upon reduction with DDT as visible in Fig. 2F), but rather a sulfonylation of the cysteine-persulfide version of C17, this was already reported previously and should be referenced [PDB ID 5LO9, Brito et al. (2016) J Biol Chem 291: 24804-24818];*

Review COMMONS

Because there was a misleading statement, as replied above, we have rewritten this paragraph. The perthiosulfonic acid (-S-SO_3^-) in Fig.2D is different from this -S-$S_2O_3^-$ in Brito et al., (2016), but consistent with Fig. 2G. This point is included in the text and the suggested paper has been cited, as requested. (lines 191-193)

> *5. Section "Crystal structure of the YeeE-YeeD complex" should be re-written. Not only it is confusing, but also undermines the tremendous amount of work done by the Authors. Please state clearle what was crystallized, how and why. Specify clearly the mutation introduced and complement Table 1 with this information;*

Thank you for these comments. The determination of the structures was certainly challenging. We have restructured the first part of the section entitled "Crystal structure of the YeeE-YeeD complex". We have included a comprehensive explanation of the crystallization process and the construction of YeeE-YeeD. Additionally, we have updated Table 1 to provide more detailed information on the two structures.

> *6. Lines 403-407: are the crystallization conditions already cryo-protected or no cryo-protection was added before flash freezing? Please state clearly;*

In response to your feedback, we have added the missing information in *MATERIALS AND METHODS* section.

> *7. Table 1:*
> *a. Is the multiplicity of PDB ID 8K1R correct? Is it really 321?? If so, is there any radiation damage to the crystal? If not, how?? Fine-fine-slicing during data collection, big crystals with elliptical data collection?? Pleas elaborate;*

The multiplicity for PDB ID 8K1R is correct. We have provided detailed information on data collection in *MATERIALS AND METHODS* section.

> *b. There are water molecules in the structure so please report number of atoms and B-factors for waters ("Solvent"), and ligands (e.g., thiosulfate, or others, if any), separately;*

We have updated Table 1 to include the requested information.

> *c. Please provide validation statistics for the structures, namely, rotamer outliers, clashscore and MolProbity score.*

We have added the validation statistics to Table 1.

> *MINOR CONCERNS*
> *1. Always reference paper and PDB ID for all structures. E.g., at line 181, only the paper is referenced;*

We have ensured that all structures are properly referenced with both the paper and the corresponding PDB ID (lines 246, 250).

> *2. Remove "alpha" in line 199;*

We have removed the "alpha" (line 268).

> *3. Add units to all concentrations. E.g., at lines 326 and 327, (w/V) and (V/V) are missing.*

We have incorporated concentration units, (w/v) or (v/v), for percentages in the appropriate locations.

> *Reviewer #3 (Significance (Required)):*
> *The scientific rationale is robust and the experimental approach is adequate and provide support to the conclusion drawn. However, there are some questions this Reviewer would like to see clarified, namely on the data collection and processing of PDB ID 8K1R.*

We appreciate your feedback. These revisions enhance the clarity and accuracy of this manuscript.
